# Improving milk donation behavior through an educational intervention based on the theory of planned behavior: study protocol of a cluster randomized controlled trial

**DOI:** 10.1186/s13006-023-00589-7

**Published:** 2023-09-19

**Authors:** Shirin Shahbazi Sighaldeh, Maryam Moridi, Vafa Ghorban Sabagh, Anoshirvan Kazemnejad, Fatemeh Shateranni

**Affiliations:** 1 Midwifery and Reproductive Health Department, School of Nursing and Midwifery, Tehran University of Medical Sciences, Tehran, Iran; 2 Breastfeeding research center-family health institute, Tehran University of Medical Sciences, Tehran, Iran; 3 Maternal-Fetal-Neonatal Research Center, Tehran University of Medical Sciences, Tehran, Iran; 4 Department of Biostatistics, School of Medical Sciences, Tarbiat Modares University, Tehran, Iran

**Keywords:** Milk banks, Donor milk, Knowledge, Attitude, Breastfeeding, Infant, Health education

## Abstract

**Background:**

Breastfeeding mothers’ decisions to donate their milk is influenced by their knowledge, attitudes, and subjective norms such as their family’s opinions on milk donation. In Iran, women have favorable knowledge and positive attitudes toward breastfeeding, but they lack sufficient information and education about milk banks. With respect to current childbearing policies, an increase in the number of infants who will be admitted to neonatal intensive care units is expected. Accordingly, improving milk donation behavior, which requires mothers’ intention and ability to donate breast milk, is important for infants’ survival and well-being. Therefore, this study aims to evaluate whether an educational program based on the theory of planned behavior affects breastfeeding mothers’ decisions to donate their breast milk.

**Methods:**

This cluster randomized controlled trial will be conducted in health centers affiliated to Tehran University of Medical Sciences and will enroll 66 breastfeeding mothers (intervention, *n* = 33; control, *n* = 33). After collecting baseline data, the intervention group will begin receiving a 60-minute weekly educational program based on Theory of Planned Behavior components for four weeks. The first follow-up assessment will be conducted immediately after the intervention, and the final one will be conducted 12 weeks later. The primary outcomes are the number of mothers who have donated their breast milk and changes in breastfeeding mothers’ knowledge, attitude, subjective norms, and perceived behavioral control about donor human milk and intention to donate milk.

**Discussion:**

This trial will evaluate if a well-designed educational program can improve breastfeeding mothers’ knowledge and promote their behavioral factors regarding milk donation and lead to a significant increase in the number of potential milk donors.

**Trial registration:**

irct.ir (IRCT20230124057203N1) registered February 14, 2023.

## Background

Exclusive breastfeeding for about the first six months and continued breastfeeding with complementary foods up to two years or more are needed to ensure optimal growth for all infants [1]. Infant feeding practices can consist of breast milk, formula feeding, and supplementary feeding [2–4]. Among these options, mother’s own milk (MOM) is uniquely designed for infants and has many health benefits for both mother and baby [5]. However, if the MOM is not available for any reason, donor human milk (DHM) available in human milk banks (HMBs) is the best option [6]. All over the world, there are more than 750 HMBs, and according to the latest updates, a total of 12 HMBs have been established in Iran [7]. The first HMB in Iran was established in 2016 at Al-Zahra teaching hospital in Tabriz, and currently, two hospitals have HMBs in Tehran (the capital of Iran) [7, 8]. One of the most important challenges for HMBs is increasing the number of donors in order to meet growing needs for donor milk because increasing donation allows more infants, who would be otherwise unable to do so, to take advantage of human milk [9].

Women’s decisions to donate their milk is influenced by their knowledge, attitude and subjective norms like their husband’s opinions [10, 11]. A descriptive study of the experiences of women who donated their milk to a HMB showed that donors had strong positive attitudes toward breastfeeding, and 96% of them felt that they were knowledgable about milk donation. Besides, participants reported that they felt supported by their husband, family and friends in different ways, and none of them reported feeling unsupported. In this study women described their confidence and ability to donate their milk which indicate the role of control beliefs in their milk donation experience [12].

In 2019, the Human Milk Banking Association of North America reported that nearly 7.4 million ounces of pasteurized human milk were donated by 12,491 breastfeeding mothers representing a nearly one million ounce increase over the number of ounces donated in 2018 [13]. In Iran in 2020, it was reported that 7,270 ounces of human milk were donated by 81 mothers, and in 2021, 28,167 ounces of human milk were donated by 51 mothers in the Omolbanin HMB (the fourth HMB in Iran) [14]. Considering that the average age of marriage in Iran has increased and the government is prioritizing childbearing policies, there are many mothers who become pregnant at advanced ages, and these mothers are at a higher risk of experiencing pregnancy complications that can result in premature birth [15]. Therefore, it is expected that the number of infants who are admitted to neonatal intensive care units will increase and, increasing the number of milk banks and improving attitudes toward milk donation is crucial in ensuring the survival and well-being of these infants.

Reviewing the literature on milk donation behavior has revealed that mothers often do not have enough knowledge about milk donation and HMBs, and especially in Iran, despite the favorable knowledge and positive attitudes of women toward breastfeeding, the availability of information and education about milk bank to mothers is very limited. Therefore, there is a need for educational interventions by health professionals to increase mothers’ knowledge and promote factors that influence milk donation behavior, and to encourage potential donors to donate their breast milk to HMBs [10–12, 16, 17].

Meanwhile, in regard to breastfeeding and milk donation, it seems that behavioral theory-based educational interventions have the ability to provide a useful framework to give essential information to mothers and improve their understanding on this subject [12, 18]. One such behavioral theory is the theory of planned behavior (TPB), which is commonly used to understand how human behavior works [19]. Since milk donation is a voluntary behavior that requires the mothers’ intention and ability to donate their breast milk, the most matching theory to this behavior and this study’s objectives is TPB. Thus, this study seeks to assess whether the educational program based on theory of planned behavior (TPB) influences breastfeeding mothers’ decision to donate their breast milk.

### Hypothesis

An educational intervention based on TPB on breastfeeding mothers will produce changes in knowledge, attitude, subjective norms, perceived behavioral control, and intention of donating breast milk which in turn will modify behavior, thus resulting in increased breast milk donation to HMBs.

### Objectives

The main objective of this study is to assess the effectiveness of an educational intervention based on TPB in breastfeeding mothers on donating breast milk to HMBs.

Secondary objectives of the study are:

1. To evaluate changes in breastfeeding mothers’ knowledge about milk donation.
2. To evaluate the effect of the educational program on behavioral determinants (attitude, subjective norms, perceived behavioral control and behavioral intention).
3. To analyze the association of behavioral determinants with milk donation.
4. To study the relationship between social demographic variables and milk donation.

## Methods

### Study design

A cluster randomized controlled trial will be carried out on breastfeeding mothers referred to health centers affiliated to Tehran University of Medical Sciences. A cluster randomized controlled trial design was chosen because it makes it easier for mothers to attend classes at their local health center, and in this design, the phenomenon of contamination between the intervention and control groups is avoided. Besides, it can help us increase the representativeness and generalizability of our study population and results. The control group allows us to analyze the direct effect of the intervention and discount an increase in milk donation due to other factors [20]. This study was approved by the Research Ethics Committees of School of Nursing and Midwifery & Rehabilitation - Tehran University of Medical Sciences (IR.TUMS.FNM.REC.1401.150).

### Setting

This study will be conducted in health centers affiliated to Tehran University of Medical Sciences. Tehran University of Medical Sciences covers primary health care services of 5 out of 22 districts of Tehran City (district numbers 10, 11, 16, 17 and 19) [21]. These health centers were chosen because they cover a significant portion of breastfeeding mothers of South Tehran, who usually bring their infants to theses\ health centers for vaccination, screening or growth monitoring. However, we will include four of these districts in our study (district numbers 10, 11, 16 and 17) because the health centers of district number 19 do not have the equipment for holding in-person classes and the population of Iranian breastfeeding mothers in that district is limited. These four districts have a total of 20 health centers. From among all these health centers, we will choose eight centers using simple randomization method; then, a cluster of four centers will be randomly selected for collecting the intervention group and four centers for collecting the control group. We will recruit eight to nine participants from each health center.

### Recruitment

Eligibility for the study is as follows: being breastfeeding mothers who are within one year after giving birth and have sufficient breast milk for their own baby, with the approval of a doctor or midwife, having no serious medical condition (both mother and infant), and being a resident of Tehran.

The exclusion criteria are: breastfeeding cessation, loss of baby, not participating in more than one session of educational program, and not responding to more than 20% of the questionnaire.

Breastfeeding mothers who meet the inclusion criteria will be selected by convenience sampling, and they will be approached to explain the study aims, risks and benefits in a way that is easily understood. If they give permission to participate in study, they will provide written informed consent, then they will fill out the pretest questionnaire in order to obtain the baseline assessment.

### Sample size

In order to provide a power of 95% with 95% confidence interval the following formula and the standard deviation of 3.17 to 3.21 (according to the same study’s findings) will be used [18]. The sample size was estimated at 30, and then allowing for 10% attrition, we plan to recruit 33 samples per group and a total of 66 study participants.

$$ n=\frac{{\left({z}_{1-\frac{\alpha }{2}}+{z}_{1-\beta }\right)}^{2}\left({s}_{1}^{2}+{s}_{2}^{2}\right)}{{\left({\overline{x}}_{1}-{\overline{x}}_{2}\right)}^{2}}$$

$$ \frac{{\left(1.96+1.64\right)}^{2}\left({3.21}^{2}+{3.17}^{2}\right)}{{3}^{2}}=29.4\cong 30$$

$$ 30+30\left(0.1\right)=33$$

### Development of the educational intervention

In this study, the educational intervention is designed by reviewing literature comprising guidelines and statements on donor milk, human milk bank, storage and preparation of breast milk and the importance of breast milk from the following sources: Human Milk Banking Association of North America (HMBANA), European Milk Bank Association (EMBA), World Health Organization (WHO), United Nations Children’s Fund (UNICEF), Centers for Disease Control and Prevention (CDC), and American Academy of Pediatrics (AAP) [22–24]. We also used the Human Milk Bank Clinical Guideline in Iran, which covers various aspects such as the infrastructure, awareness, communication, ethical issues, donors, recipients, and working processes of a human milk bank [25].

After that, we applied the Delphi method to gather and validate the material according to the opinions of experts in midwifery and reproductive health and neonatology of Tehran University of Medical Sciences. We conducted three rounds of questionnaires with the panel, which consisted of three neonatologists, three midwives who have experience in breastfeeding education, one health education professional, and one midwife who was in charge of a milk bank. They were well informed and familiar with the Iranian culture and community, especially regarding the issues of milk kinship and the role of the husband and families in Iranian mothers’ milk donation, and breastfeeding mothers’ skills and self-efficacy in expressing their breast milk.

### Intervention design

Following baseline data collection, the intervention group will start receiving 60-minute weekly educational intervention based on TPB components for four weeks (Table 1).

**Table 1:** The content of the educational sessions based on the theory of planned behavior

Session	Main purpose	Educational materials	Educational methods and time schedule
1	Improving breastfeeding mothers’ knowledge and attitudes toward DHM and HMB	• PowerPoint presentation • Booklet (including contents of all sessions) • Pamphlet of the first session	1) Introducing presenter and participants and explaining study aims (10 min) 2) Giving information on importance and background of breast milk donation and HMBs (40 min) 3) Question and answer (10 min)
2	Addressing the role of subjective norms in breast milk donation	• PowerPoint presentation • Pamphlet of the second session	1) Reviewing previous session (5 min) 2) Giving information related to Islamic laws of milk donation (15 min) 3) Presenter will use the psychodrama method. In this method two volunteer breastfeeding mothers role-play problems and release their intense emotions in regard to milk donation, then the presenter will guide their subjective norms [26] (30 min) 4) Question and answer (10 min)
3	Addressing the role of perceived behavioral control in breast milk donation behavior	• PowerPoint presentation • Pamphlet of the third session • Video	1) Reviewing previous session (5 min) 2) Giving information on requirements for breast milk donation, indication and contraindication of breast milk donation, process and cost of HMBs and current status of HMBs in Tehran (40 min) 3) Screening a video which shows how a HMB works (5 min) 4) Question and answer (10 min)
4	Addressing the behavioral intention and explaining how to implement the behavior of donating breast milk	• PowerPoint • Pamphlet of the fourth session • Video • Breast pump	1) Reviewing previous session (5 min) 2) Giving information on breast milk expression (10 min) 3) Showing how to express breast milk by hand and breast pump (25 min) 4) Showing videos of successful breast milk expression (10 min) 5) Question and answer (10 min)

Lectures, small groups discussion, educational pamphlets and booklet, PowerPoint presentations, showing videos and pictures, will be the main educational methods and materials that we are going to use in order to make the program effective and interesting. Moreover, we will ask mothers to share the booklet and pamphlets with their family and friends. Sessions will be held in health centers for the intervention group while the control group will receive only usual education.

### Data collection

The questionnaire will be uploaded to Porsline, a well-known online survey platform in Iran, to create a link for the questionnaire. Participants can use their mobile phones to open the link and fill out the questionnaire. A baseline assessment will be conducted prior to the intervention for both the intervention and control groups. In order to assess the immediate and long-term effects of the intervention prospectively, the first follow up assessment will be conducted immediately after the intervention, and the final one will be conducted 12 weeks later. Our primary outcomes are the number of mothers who have donated their breast milk and changes that have happened in breastfeeding mothers’ knowledge, attitudes, subjective norms, perceived behavioral control about DHM and intention to donate milk. Our secondary outcomes are the associations of behavioral determinants and social demographic variables with milk donation.

### Development of the measurement tool

Data will be measured by a 46-item researcher-made questionnaire. This instrument has been created by reviewing literature on milk donation and previous studies on TPB and consists of seven sections as described in Table 2.

**Table 2:** Theory of planned behavior-based survey questions

Sections	Example of questions	Scale
Sociodemographic and perinatal characteristics	Age, education, milk and blood donation history, breastfeeding history, economic status, parity, mode of delivery, the gestational age, sex and age of infant, awareness of HMBs and the source of information of HMBs	-
Knowledge of DHM and HMBs	“What is a human milk bank?” “What is the best alternative to mothers’ own milk for feeding premature babies?” “What effects does pasteurization have on donor milk?” “In the hospital, which babies receive donor milk?” “Who is eligible to donate their breast milk?” “How is donor milk stored in the hospital?” “How long can donated breast milk be stored in the hospital?” “What effects has pasteurized donor milk on premature babies?” “What should mothers do before donating their breast milk?” “What are the characteristics of informal breast milk sharing in the community?”	Multiple-choice questions
Attitude toward DHM and HMBs	“It is pleasant for me to donate breast milk” “Feeding premature babies with donor milk is better than formula” “Donor milk can reduce the stress of mothers who can’t feed their babies with their own breast milk” “By donating breast milk to a milk bank, I can save a baby’s life” “Donating breast milk to the milk bank can reduce the incidence of serious gastrointestinal disease in infants” “Donor milk in milk banks is safe and healthy” “When I don’t have enough breast milk, it is better to use donor milk instead of formula to feed my baby” “Donor milk in a milk bank may carry viruses and spread disease” “Donor milk should be used widely in hospitalized infants who cannot be breastfed, if it is possible” “Donor milk can treat newborns who weigh less than normal” “I think mothers should be rewarded for donating their milk” “After pasteurization, the nutrients in milk are all destroyed, so it is better to feed baby with formula”	5-point Likert (from strongly agree to strongly disagree)
Subjective norms about DHM and HMBs	“My husband’s opinion on donating milk to the milk bank is important to me” “I will not donate my breast milk to the milk bank if my husband disagrees” “Opinions of people around me like my mother, family, friends etc. on donating milk are important to me” “I will not donate my milk to the milk bank if people around me (like my mother, family, friends etc.) disagree” “Milk donation and using donor milk have ethical issues in Islam” “God considers milk donation as a virtue and admirable action” “If I want to donate my milk, my family will support me”	5-point Likert (from strongly agree to strongly disagree)
Perceived behavioral control in milk donation	“I feel donating my milk will decrease my milk supply which is needed for my own baby” “It costs me a lot to donate my milk” “I can provide equipment for milk donation such as a container for storing milk, breast pump (if needed)” “I think I can be a milk donor” “If my husband or family are against donating milk, I can convince them” “I can express my breast milk correctly” “I can provide storage conditions for expressed milk”	5-point Likert (from always to never)
Intention to donate milk	“I am going to donate my milk to a milk bank within the next six months” “I am going to identify the milk banks where I live” “I am going to contact a milk bank within the next six months” “I am going to identify the nearest route to the milk bank” “I am going to undergo a pre-donation blood test” “I am going to inform other breastfeeding mothers about milk banks” “I am going to provide equipment for milk donation”	5-point Likert (from always to never)
Breastmilk donation	“I donated my breast milk in the last six months” “How many times did you donate your milk to the milk bank in the last six months?” “I will follow hygiene tips if I want to donate my breast milk”	Yes / No Number 5-point Likert (from always to never)

Theory of planned behavior assumes that individuals’ behavior is directly determined by three main components, namely: attitude, subjective norms, and perceived behavioral control [18, 19].

- Behavior: the action of donating breast milk.
- Intention: perceived likelihood of donating breast milk.
- Attitude: favorable or unfavorable perceptions of donating breast milk which are determined by breastfeeding mothers’ knowledge, beliefs, and experiences.
- Subjective norms: beliefs about whether most people who are important to breastfeeding mothers approve or disapprove of donating breast milk.
- Perceived behavioral control: breastfeeding mothers’ perceptions of the ease or difficulty of donating breast milk.

The reliability of questions will be checked for internal consistency (Cronbach’s alpha) and the content validity of the questionnaire will be assessed by both qualitative and quantitative methods. In the qualitative method, ten experts in breastfeeding and the health promotion field will assess questioners. The experts include Faculty members of the Midwifery and Reproductive Health Department and neonatologists of Tehran University of Medical Sciences, and, after receiving their opinions, any required modification will be made. In the quantitative method, content validity index (CVI) and content validity ratio (CVR) will be measured. For face validity we will ask ten breastfeeding mothers to provide feedback on the clarity of the questionnaire and based on their opinions some essential clarification will be made.

### Statistical analysis

We will use descriptive analysis to report baseline characteristics. In this study the Repeated ANOVA between subjects test will be used for quantitative variables which have a normal distribution, and the Friedman test will be used for quantitative variables that do not have a normal distribution. For qualitative variables, chi-square test or Fisher’s exact test will be used. The effectiveness of the intervention will be assessed using the repeated measure ANOVA test and the effect of demographic variables on milk donation behavior will be determined using a logistic regression model. Statistical analyses will be made using IBM-SPSS 22. The trial has been registered at the Iranian Registry of Clinical Trials (IRCT20230124057203N1).

## Discussion

A well-designed educational program is crucial to improving breastfeeding mothers’ knowledge and behavior regarding milk donation. Especially in Iran, some Muslim mothers may potentially avoid using DHM or donating their breastmilk because they may wrongly assume that milk donation can lead to milk kinship between infants or is not allowed in Islam. However,milk donation is definitely allowed and is even praised as a virtue in Islam, so they need to be informed about Islam Sharia law related to milk donation [17]. In addition, Iranian mothers have a positive attitude towards breastfeeding and the prevalence of exclusive breastfeeding among them is at a satisfactory level [27, 28]. Therefore, educating mothers can lead to a significant increase in the number of potential milk donors [27].

Upon the completion of this study, an increase in milk donation and TPB components scores may indicate that this educational intervention is useful in increasing milk donation behavior. Similarly, negative results can help us devise new strategies in order to improve milk donation behavior. Additionally, in this study we will assess which components of TPB are the most important ones in milk donation behavior, and these findings can guide the content used in future programs and studies.

## Data Availability

Not applicable.

## Abbreviations

DHM: Donor human milk
HMB: Human milk bank
MOM: Mother’s own milk
TPB: Theory of planned behavior
